# Combing Seeding Crystallization with Flotation for Recovery of Fluorine from Wastewater: Experimental and Molecular Simulation Studies

**DOI:** 10.3390/molecules28114490

**Published:** 2023-06-01

**Authors:** Hao Zhang, Jue Kou, Chunbao Sun

**Affiliations:** School of Civil and Resource Engineering, University of Science and Technology Beijing, Beijing 100083, China

**Keywords:** seeding crystallization, flotation, fluorine, recovery, wastewater

## Abstract

For effective removal and utilization of fluorine resources from industrial wastewater, stepwise removal and recovery of fluorine were accomplished by seeding crystallization and flotation. The effects of seedings on the growth and morphology of CaF_2_ crystals were investigated by comparing the processes of chemical precipitation and seeding crystallization. The morphologies of the precipitates were analyzed by X-ray diffraction (XRD) and scanning electron microscope (SEM) measurements. The seed crystal, fluorite, helps improve the growth of perfect CaF_2_ crystals. The solution and interfacial behaviors of the ions were calculated by molecular simulations. The existing perfect surface of fluorite was proven to provide the active sites for ion adhesion and formed a more ordered attachment layer than the precipitation procedure. The precipitates were then floated to recover calcium fluoride. By stepwise seeding crystallization and flotation, the products with a CaF_2_ purity of 64.42% can be used to replace parts of metallurgical-grade fluorite. Both removal of fluorine from wastewater and the reutilization of the fluorine resource were realized.

## 1. Introduction

As phosphate ores which often contain fluoride are being used, large amounts of wastewater containing fluoride are produced in the phosphate industry [1,2]. The high-level fluorine in water is a toxic substance to the human body that can cause dental fluorosis, crippling deformities, osteoporosis, osteosclerosis, and cancer [3,4]. It can also ruin the normal growth and development of children’s brains and minds. In view of the hazards of fluoride, there are strict requirements for the discharge of industrial wastewater [5,6]. In China, the permissible limit of fluoride for discharge is 10 mg/L [7].

To meet the discharge requirements, numerous methods have been researched to remove fluoride from industrial wastewater [8]. Traditional technologies, such as chemical precipitation and adsorption, present the advantages of high removal efficiency, low cost, and simple operation [9]. However, the low-quality sludge prevents the application of chemical precipitation at a large scale [10], and the additional cost caused by dissolution loss and regeneration of the adsorbent limits the practical application of the adsorption in fluoride removal [11]. Then, alternative methods, including reverse osmosis, ion exchange, membrane, and electrodialysis, have been used [12]; however, in addition to the high costs, these methods have the disadvantages of difficulties in recycling, solid waste pollution, and waste of resources [13,14].

Despite the drawbacks of the generation of large amounts of sludge with high water content, chemical precipitation still serves as a common method in industrial wastewater treatment [15]. The crystallization determines the characteristics of the sludge produced in the chemical precipitation process [16]. To obtain valuable secondary products for further recovery, seeding crystallization, which is a reliable technique for improving the controllability of crystallization, is introduced to the chemical precipitation [17,18]. It can reduce the complexity of crystallization and save time. Thus, both high efficiency and qualified products can be obtained by adding seedings in the chemical precipitation process.

For the sustainable development of the environment and resources, the removal and simultaneous recovery of useful substances from wastes attract more and more attention [19]. Fluoride compounds, which mainly come from natural resources, are important industrial raw materials. Calcium fluoride (CaF_2_) is one of the main materials with a wide range of applications, based on which it is commercialized as acid-grade, ceramic-grade, and metallurgical-grade [20]. To deal with the high consumption of natural resources, the recovery of fluoride from waste is considered a priority. Thus, both the removal and recovery of fluorine should be involved in the subsequent applications [21]. Currently, flotation is the most common physical beneficiation process to purify and recover valuable substances from solid matter [22,23]. It is also the most frequently used method to recover natural fluorine resources, fluorite [24,25].

In this study, an innovative stepwise method was performed. Both removal and recovery of fluorine from industrial wastewater were realized using seeding crystallization and flotation. Seedings were added during the crystal process to ensure the high flotation recovery of fluorine from wastewater in a subsequent step. The main objective of the present study was to explore the growth of CaF_2_ crystal with fluorite seedings and the subsequent recovery of the crystal by flotation. Analyses of both crystal morphology and molecular dynamics simulation were carried out.

This study could provide theoretical references for crystal growth, as well as technical storage and support for industrial wastewater treatment and resource utilization. Large amounts of industrial wastewater containing fluoride can be reused with the environmental impact of pollutants reduced [26], and the environment and resource sustainability can be attained. In addition, fluorine could be recovered and turned into useful fluorine products. It brings both economic and environmental benefits. Furthermore, the sustainable production and development of enterprises can be ensured.

## 2. Results and Discussion

### 2.1. Removal of Fluorine

#### 2.1.1. Removal of Fluorine by Chemical Precipitation

**Figure 1:**
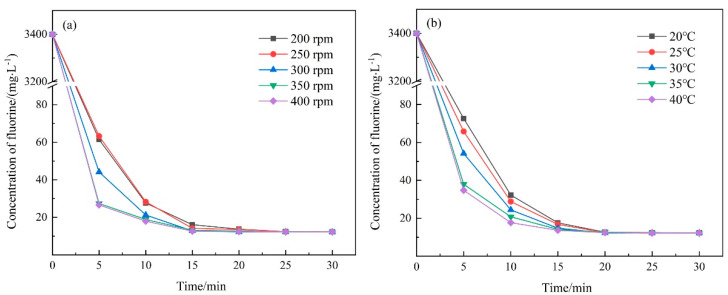
Changes in fluorine concentration at different (**a**) stirring speeds and (**b**) temperatures.

For the high-efficiency removal of fluorine, chemical precipitation with calcium hydroxide was carried out. The effects of stirring speed and temperature on the removal of fluorine were explored to obtain the optimal precipitation condition. The results are displayed in Figure 1. The concentration of fluoride in water was detected to represent the precipitation of fluorine.

As shown in Figure 1a, under the explored stirring speed, the residual concentration of fluorine in water was reduced to less than 13 mg·L^−1^ within 20 min. In the first 5 min, the reduction rate of fluorine increased with the increasing stirring speed. However, for the precipitation between 5 and 15 min, the reduction rate decreased with the increasing stirring speed. When the stirring speed was set at 300 rpm, the reduction rate of fluorine was moderate in the whole stirring test. The residual concentration of fluorite decreased rapidly to 12.25 mg·L^−1^ after 15 min, but it took 20 min for the stirring speed of less than 300 rpm. Thus, the stirring speed of 300 rpm was chosen for the precipitation tests. Changes in fluorine concentration at different temperatures, displayed in Figure 1b, showed a similar tendency to those at different stirring speeds. In the first 5 min, the reduction rate of fluorine increased with the increasing temperature. For the precipitation between 5 and 15 min, the reduction rate decreased with the increasing temperature. Based on the evaluation of both removal efficiency and cost, a room temperature close to 25 °C and a 20 min precipitation time were adopted for further investigations.

**Figure 2:**
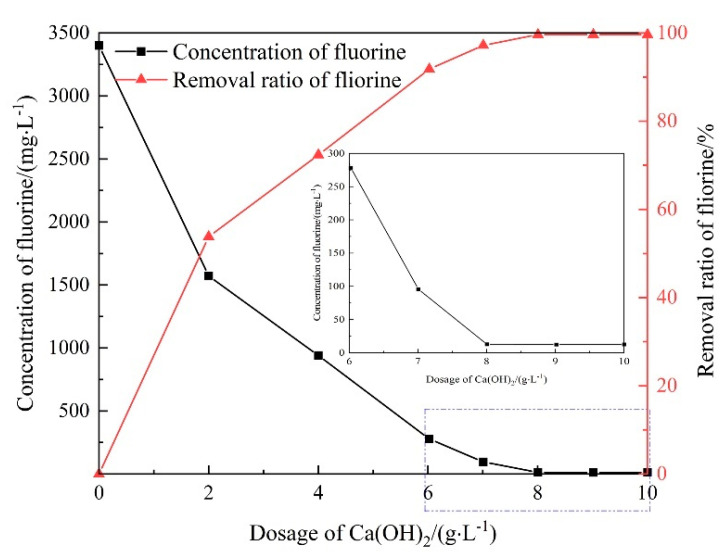
Changes in fluorine concentration at different Ca(OH)_2_ dosages.

Figure 2 displays the removal of fluorine with different dosages of Ca(OH)_2_ in chemical precipitation. The removal ratio of fluorine increased with the increasing dosage of Ca(OH)_2_ and then remained stable when the dosage of Ca(OH)_2_ exceeded 8 g·L^−1^. The residual fluorine concentration was 12.61 mg·L^−1^ by adding 8 g·L^−1^ of Ca(OH)_2_. The removal ratio reached 99.64%. However, the lowest fluorine concentration of the water after chemical precipitation was approximately 12 mg·L^−1^, which is still higher than the permissible limit.

#### 2.1.2. Removal of Fluorine by Seeding Crystallization

**Figure 3:**
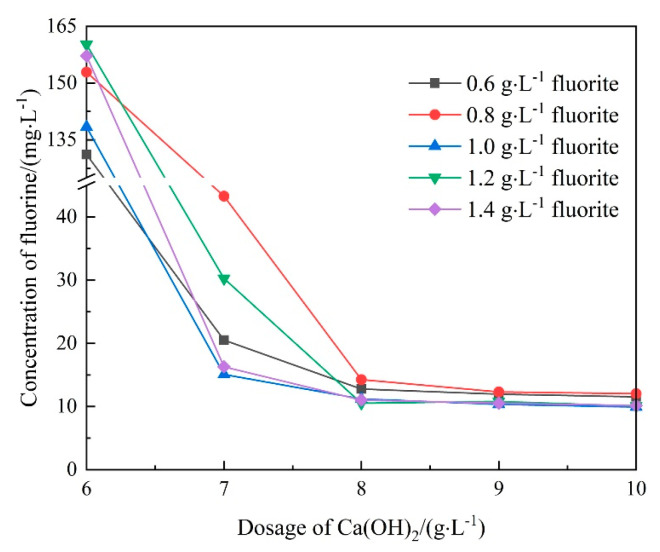
Changes in fluorine concentration at different fluorite dosages.

To meet the drainage standard, seeding crystallization was used in the simulated wastewater treatment to lower the final fluorine content. Fluorite acted as the crystal seed and was added to the precipitation system. The changes in fluorine concentration at different fluorite dosages are presented in Figure 3. The residual concentration of fluorine in water fluctuated with the increasing fluorite dosage. With lower fluorite amounts of 0.6 and 0.8 g·L^−1^, the residual concentration of fluorine was 11.53 and 12.03 when 10 g·L^−1^ of Ca(OH)_2_ was added. However, with higher fluorite amounts of 1.0, 1.2, and 1.4 g·L^−1^, the residual fluorine concentration decreased to approximately 10 mg·L^−1^, which meets the drainage standard. Thus, the precipitation of the fluorine was improved by fluorite.

#### 2.1.3. Analysis of Precipitates

**Figure 4:**
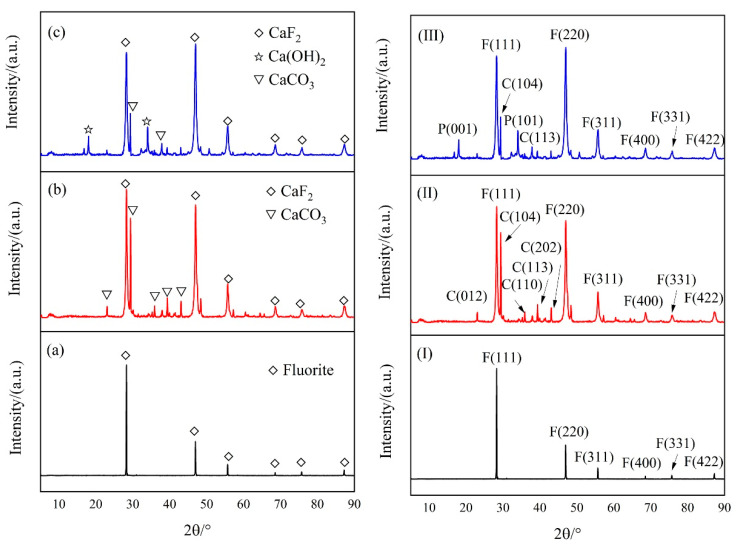
XRD patterns of fluorite (**a**,**I**), precipitates with fluorite (**b**,**II**) and Ca(OH)_2_, and precipitates with Ca(OH)_2_ (**c**,**III**). F: fluorite, C: calcite, P: portlandite.

The main phase composition and crystal plane of the precipitates and seeds were detected and are displayed in Figure 4. For chemical precipitation, the main composition of the precipitates was CaF_2_, Ca(OH)_2_, and CaCO_3_. The main planes of CaF_2_, including (111), (220), (311), (400), (331), and (422), were detected. However, the main composition of the precipitates in seeding crystallization was CaF_2_ and CaCO_3_. The main planes were consistent with those in chemical precipitation. Ca(OH)_2_ was not detected, indicating that the seed crystal enhances the formation of CaF_2_ crystal. Then, dissolution of Ca(OH)_2_ was enhanced with the decreasing calcium concentration.

**Figure 5:**
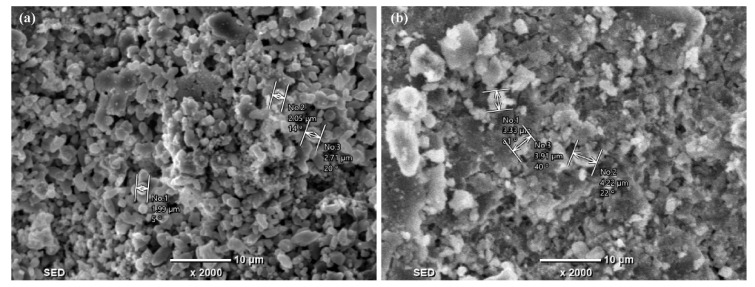
Morphology of (**a**) precipitates with fluorite and Ca(OH)_2_ and (**b**) precipitates with Ca(OH)_2_.

The morphology of the precipitates is displayed in Figure 5. For the precipitates shown in Figure 5a, perfect crystals with the distinct plane and even sizes could be easily distinguished, indicating that the seed crystal (fluorite) helps the growth of the perfect CaF_2_ crystals. In Figure 5b, the amorphous precipitates are distributed in an inhomogeneous form and irregularly. The poor crystallization of CaF_2_ in chemical precipitation will lead to the poor recovery of CaF_2_.

The attachment energy means the energy released during the attachment of the growth unit on the crystal plane [27]. According to the Gibbs thermodynamic principle and the Wulff shape of equilibrium crystals, the shape of the crystal is determined by the relative distance between the crystal center and the corresponding crystal plane [28]. For an equilibrium crystal, the distance is proportionate to the growth rate of the crystal plane, and the growth rate of the crystal planes determines the morphology of the crystal. Thus, the plane with the lowest attachment energy grows the most slowly and presents the most significant morphological characteristics [29].

Based on the Mph Growth module, the growth and morphology of the crystal were predicted. The main crystal surfaces and their attachment energy are displayed in Table 1. The simulated result is remarkably consistent with the XRD patterns shown in Figure 4. The attachment energy of the (111) plane was the lowest. Thus, (111) grows the most slowly and is the largest surface remaining during the crystal growth. The ascending order of attachment energy and growth rate is (111), (220), (311), (420), (422), (200). As the growth rate of (200) is relatively higher, the (200) plane grew rapidly and disappeared in XRD measurement, and the (400) plane was detected and displayed.

### 2.2. Solution and Interfacial Behaviors of Ions

#### 2.2.1. Solution Behaviors of Ions in Chemical Precipitation

**Figure 6:**
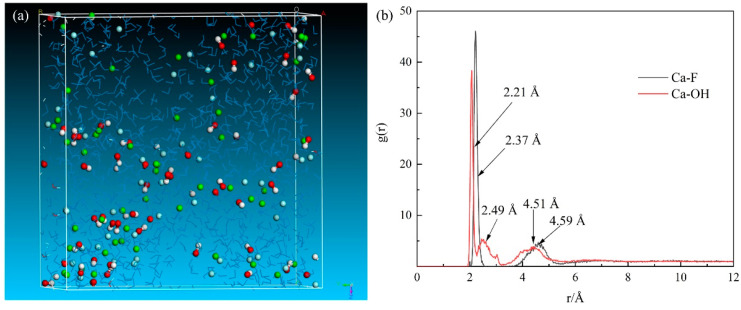
Distribution of ions in chemical precipitation: (**a**) equilibrium configuration; (**b**) radial distribution functions of Ca–F and Ca–OH. Ca: green ball, F: cyan ball, O: red ball, H: white ball, water: blue line.

**Figure 7:**
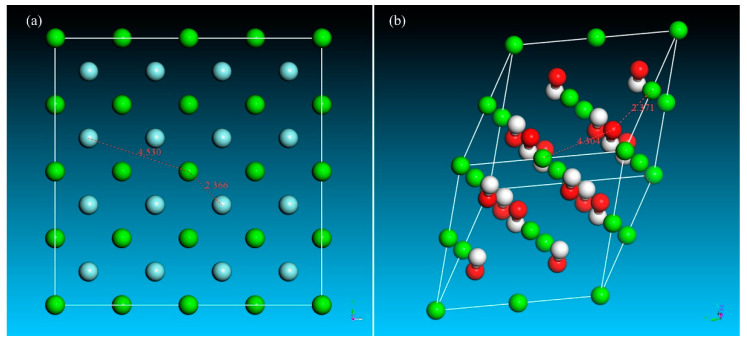
Crystal cells of (**a**) fluorite and (**b**) calcium hydroxide.

The solution behavior of ions, including movement, collision efficiency, and distribution in water, determines the chemical precipitation of fluorine. The equilibrium solution system containing water molecules and ions that participated in precipitation was calculated to analyze the solution behavior of the ions. As shown in Figure 6, the randomly and disorderly distributed ions tended to the relatively orderly accumulated due to the precipitation reaction. The nearest fluorine ions were distributed approximately 2.37 Å from the calcium ions. The distance equals the distance between calcium and fluorine in fluorite crystal shown in Figure 7a. Another peak of Ca–F is located at 4.59 Å and is close to the distance between calcium and sublayer fluorine. It could induce a fluorite crystal to form.

The three peaks in the radial distribution function of Ca–OH showed that the distances between calcium and hydroxyl ions were 2.21, 2.49, and 4.51 Å. However, as shown in Figure 7b, the distances between calcium and hydroxyl ions in the calcium hydroxide crystal are 2.37 and 4.34 Å for the nearest and the sublayer atoms, respectively. The first peak located near 2.21 Å refers to the freely distributed hydroxyl ions, and the hydroxyl ions distributed at the ranges from 2.37 to 3.00 Å and 3 to 5 Å indicate the interaction between calcium and hydroxyl ions. The ill-defined peaks for the formation of calcium hydroxide crystal illustrate that the formation of fluorite crystal has priority. Thus, the chemical precipitation by adding calcium hydroxide can be realized.

#### 2.2.2. Interfacial Behaviors of Ions in Seeding Crystallization

**Figure 8:**
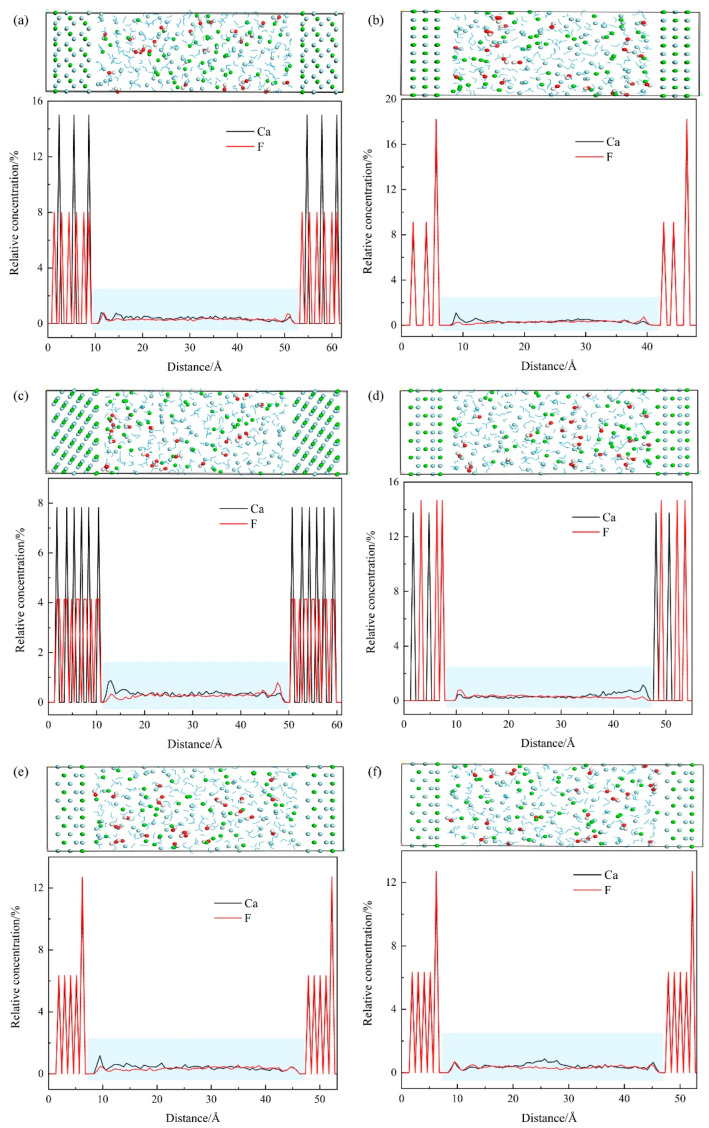
Distribution of calcium and fluorine on different fluorite surfaces: (**a**) (111); (**b**) (220); (**c**) (311); (**d**) (400); (**e**) (331); (**f**) (422).

The behavior of ions on the crystal plane plays a vital role in crystal growth. For seeding crystallization, the existing crystal planes provided by seed crystal save the nucleation energy [30]. The surface behavior of calcium and fluorine on the exposed crystal face of fluorite was calculated and analyzed based on the equilibrium reaction configurations. The attachment configuration and the relative concentration of ions in the system are displayed in Figure 8. The distance peaks of calcium and fluorine located near the fluorite surfaces indicate the attachment of the ions on these surfaces.

The difference in the growth rate of the plane results in the different morphology and growth rate of the crystal [31,32]. Compared to the chemical precipitation, the existing perfect surface of fluorite provides the active sites for ion adhesion. The growth of the crystal is induced and accelerated [33]. For the (111) surface of fluorite, the distance between the surface calcium and the sublayer fluorine is 2.62 Å. During seeding crystallization, the calcium in the solution formed the first attachment layer, which is ordered. The distance between the solution calcium and the surface fluorine is 2.62 Å and equals the distance between the surface calcium and the sublayer fluorine at the (111) surface. The distance between the solution fluorine and the surface calcium is 3.14 Å and equals the surface fluorine and sublayer calcium. Thus, a stable growth layer in parallel with (111) forms. At the (220) plane, the distance between surface calcium and sublayer fluorine is 1.61 Å, while the distance between solution calcium and surface fluorine is 3.22 Å. The loose attachment of the ions on the (220) plane refers to the low growth rate. At the (311) plane, the distances between solution calcium and surface fluorine, between surface calcium and solution fluorine, and between solution calcium and solution fluorine are 2.55 Å and are equal to the distance between surface calcium and crystal fluorine. For the (400) plane, the distance between surface fluorine and sublayer calcium is 2.52 Å. The distances between solution calcium and surface fluorine and between surface calcium and solution fluorine range from 2.52 to 3.02 Å. Combined with the analysis of the crystal surface and the XRD patterns, the indefinite adhesion layer and the longer distance indicates the unstable surface, which will decrease or even disappear with the crystal growth. At the (331) and (422) planes, the distances between the first-layer atoms and the second-layer atoms, the third-layer atoms, and the fourth-layer atoms are 1.08, 2.16, and 3.25 Å, respectively, while the distance between the solution calcium and the surface fluorine, as well as between the surface calcium and the solution fluorine, is 3.25 Å. The relatively unstable layer will decrease or disappear.

### 2.3. Flotation Recovery of Fluorine

#### 2.3.1. Flotation of Precipitates after Chemical Precipitation

**Figure 9:**
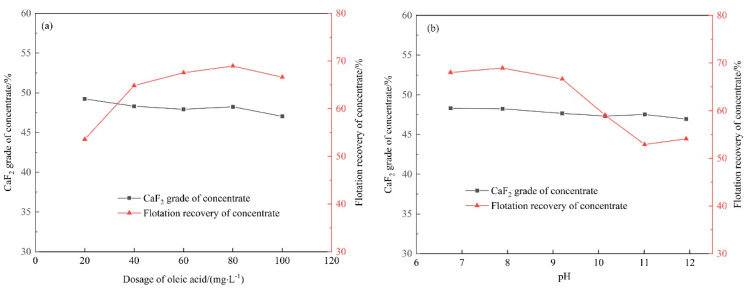
Flotation of precipitates with Ca(OH)_2_ in the function of (**a**) oleic acid dosage and (**b**) pH.

Large amounts of sludge containing CaF_2_ crystals were produced after both chemical precipitation and seeding crystallization. The recovery of CaF_2_ crystals determines the subsequent utilization of fluorine. Flotation can be used to separate CaF_2_ from other impurities by avoiding filtrating and drying the sludge. The flotation results of the precipitates after chemical precipitation are shown in Figure 9. With the increasing dosage of oleic acid, the CaF_2_ grade of the concentrate decreased while the CaF_2_ recovery increased at first and decreased when the dosage of oleic acid exceeded 80 mg·L^−1^. Then, 80 mg·L^−1^ of oleic acid was adopted for pH experiments. Both grade and recovery of CaF_2_ in concentrate showed downward trends with the increasing pH. The optimal pH of the slurry was set at 7.89. The concentrate with CaF_2_ grade of 48.24% and recovery of 68.94% was obtained.

#### 2.3.2. Flotation of Precipitates after Seeding Crystallization

**Figure 10:**
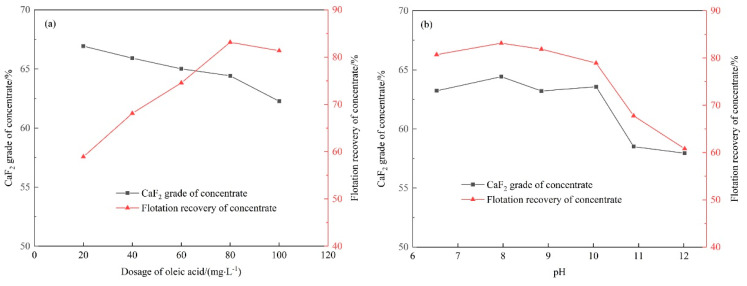
Flotation of precipitates with Ca(OH)_2_ and fluorite in the function of (**a**) oleic acid dosage and (**b**) pH.

Flotation results of the precipitates after seeding crystallization are shown in Figure 10. The changes in the grade and recovery of CaF_2_ are similar to those of flotation concentrate after chemical precipitation. The CaF_2_ grade of the concentrate decreased with the increasing dosage of oleic acid, and the CaF_2_ recovery increased at first and decreased when the dosage of oleic acid exceeded 80 mg·L^−1^. Both grade and recovery of CaF_2_ in concentrate show overall downward trends with the increasing pH. Finally, the concentrate with CaF_2_ grade of 64.42% and recovery of 83.14% was obtained. Compared to the flotation concentrate above, both the grade and recovery of the concentrate were improved. Thus, after the stepwise seeding crystallization and flotation treatment of the fluorine wastewater, the products can be used to replace parts of metallurgical-grade fluorite, with a CaF_2_ purity of 60~85% [34].

## 3. Materials and Methods

### 3.1. Materials

Sodium fluoride (NaF) of analytical grade, which was purchased from the Sinopharm Chemical Reagent Co., Ltd. of Beijing, China, was used to prepare the simulated fluorine wastewater. Deionized water was used to avoid the potential effects of ionic species in water. Based on the fluorine concentration of the industrial acid water (0.34 wt.%), the initial fluorine concentration used in the study was controlled at 0.179 mol·L^−1^, which is approximately 3400 mg·L^−1^. Calcium hydroxide (Ca(OH)_2_) of analytical grade was also purchased from the Sinopharm Chemical Reagent Co., Ltd. of China. To reduce costs, the fluorite concentrates (with a purity of 98%) were used as seed crystals, and the fluorite concentrates were obtained from the flotation of low-quality ore. Sodium hydroxide and hydrochloric acid, purchased from Sinopharm Chemical Reagent Co., Ltd., were used as pH regulators in flotation. Oleic acid of technical grade, purchased in Hengyuan Grease Chemical Plant, was used as the flotation collector.

### 3.2. Experimental Procedures

The reaction beaker equipped with a magnetic stirrer was used for the precipitation experiment. A total of 200 mL of simulated fluorine wastewater was added, followed by adding Ca(OH)_2_ and fluorite. The stirring speed, time, and temperature were studied. Afterward, the precipitates were washed and dried in an oven with the temperature controlled under 60 °C. The washing water was collected to measure the volume and the content of fluorine. The fluorine concentration was detected by a fluorine ion-selective electrode, and the residual fluorine concentration of the wastewater was calculated based on the values in the beaker and washing water. The removal ratio of fluorine was calculated by the following equation:

(1) $$R=\frac{C_{o}-C_{f}}{C_{o}}\times 100\%$$

where *C_o_* and *C_f_* refer to the original concentration and the residual concentration of fluorine in wastewater.

With the optimal precipitation conditions, the suspension was settled with the supernatant removed. The remaining suspension was then transferred to a 50 mL flotation cell equipped with an XFGⅡ flotation machine with an agitating speed of 1662 r/min. The pH was adjusted and then conditioned for 2 min. Oleic acid was added subsequently. After 2 min, a 4 min flotation was performed. Both concentrate and tailing were collected, filtrated, and dried. The weight and CaF_2_ grade of the products were analyzed.

### 3.3. Analytical Methods

The phase composition of the precipitates was measured by an X’Pert Pro diffractometer (X’Pert Pro MPDDY2094, PANalytical B.V., Eindhoven, The Netherlands) from 5° to 90° under Cu Kα radiation. The morphology of the precipitates was characterized by a JCM-6000plus NeoScope scanning electron microscope (SEM) (JEOL, Tokyo, Japan).

### 3.4. Molecular Simulation Details

Molecular dynamics simulations performed on Materials Studio (2018) were used to investigate the solution and interfacial behaviors of ions. The original crystal of the CaF_2_ (No. 0008645) was exported from the American Mineralogist Crystal Structure Database (AMCSD) [35]. Calcium and fluoride ions and water molecules were built based on the Visualizer module, and they were packed to obtain the solution boxes based on the Amorphous Cell module. For interfacial behavior investigation, different planes were cleaved and combined with the solution boxes.

To obtain the equilibrium, geometries of the solution and interface were obtained after molecular dynamics simulations under the COMPASS (condensed-phase optimized molecular potentials for atomistic simulation studies) forcefield in the Forcite module [36]. The geometry optimization procedure was performed with the Smart algorithm, and van der Waals force and electrostatic force were calculated based on the group-based and Ewald summation methods, respectively. The molecular dynamics simulation procedure included the sequential calculation in NVT (1 ps) ensemble, NVE (50 ps) ensemble, and NVT (2 ns) ensemble [37]. The first equilibration procedure in NVT ensemble was carried out at 298 K with a Velocity Scale thermostat to find the velocity for the optimization, and the last equilibration procedure in NVT ensemble was performed with a Nose thermostat. The trajectory was recorded, and the files in the last 1 ns were used for analysis.

As an important parameter to characterize the molecule and group distribution, the relative concentration was calculated. It is defined as follows [38]:

(2) $$c=\frac{n_{p}/v_{p}}{n_{t}/v_{t}}$$

where *c* is the relative concertation, *n_p_* is the molecule and group number of per unit, *v_p_* is the volume of per unit, *n_t_* is the total molecule and group number of the system, and *v_s_* is the volume of the system.

## 4. Conclusions

Stepwise removal and recovery of fluorine from wastewater were investigated by seeding crystallization and flotation, and chemical precipitation was performed to compare the removal efficiencies, as well as the morphology of products. By adding 8 g·L^−1^ of Ca(OH)_2_ as precipitant, the removal ratio of fluorine reached 99.64%, and the fluorine concentration of water was decreased to approximately 12 mg·L^−1^. However, when 1.0 g·L^−1^ of fluorite was added as seed crystals, the residual concentration of fluorine in water could be reduced to 10 mg·L^−1^, and met the permissible limit. Additionally, the precipitates of chemical precipitation were amorphous and they were distributed in an inhomogeneous form and irregularly. Seed crystals help improve the growth of the perfect CaF_2_ crystals. More perfect crystals with distinct planes and even sizes were obtained. Deep exploration by molecular simulations showed that the existing perfect crystal surfaces of fluorite provided the active sites for regular ion adhesion. The growth of the crystal was induced, accelerated, and improved. The flotation of the precipitates after seeding crystallization precipitant can recover 83.14% of CaF_2_ with a grade of 64.42%, and the products can be used to replace parts of metallurgical-grade fluorite. As a result, the fluorine in the wastewater changed into useful products. Both economic and environment benefits were obtained.

## Figures and Tables

**Table 1:** Simulated main crystal surfaces and their attachment energy.

(h k l)	E_att_/(Kcal mol^−1^)
1 1 1	−76.26
2 0 0	−1304.59
2 2 0	−301.71
3 1 1	−753.34
3 3 1	−259.54
4 2 0	−805.34
4 2 2	−813.80

## Data Availability

Not applicable.
